# Solvent Selection as a Key Factor in the Performance of Semitransparent Heterojunctions Composed of Hydrogenated Nanotubes and Bismuth Sulfides

**DOI:** 10.1021/acsami.4c18233

**Published:** 2025-01-16

**Authors:** Stefania Wolff, Wiktoria Lipińska, Justyna Gumieniak, Agnieszka Kramek, Karol Załęski, Emerson Coy, Natalia A. Wójcik, Katarzyna Siuzdak

**Affiliations:** † Advanced Materials Centre and Division of Electrochemistry and Surface Physical Chemistry, Institute of Nanotechnology and Materials Engineering, Gdańsk University of Technology, 11/12 G. Narutowicza Street, 80-233 Gdańsk, Poland; ‡ Centre for Plasma and Laser Engineering, Institute of Fluid-Flow Machinery, Polish Academy of Sciences, 14 Fiszera Street, 80-231 Gdańsk, Poland; § The Faculty of Mechanics and Technology, Rzeszów University of Technology, Kwiatkowskiego 4 Street, 37-450 Stalowa Wola, Poland; ∥ NanoBioMedical Centre, Adam Mickiewicz University, Wszechnicy Piastowskiej 3, 61-614 Poznań, Poland

**Keywords:** TiO_2_ nanotubes, bismuth sulfide, photoelectrochemical activity, SILAR, IPCE

## Abstract

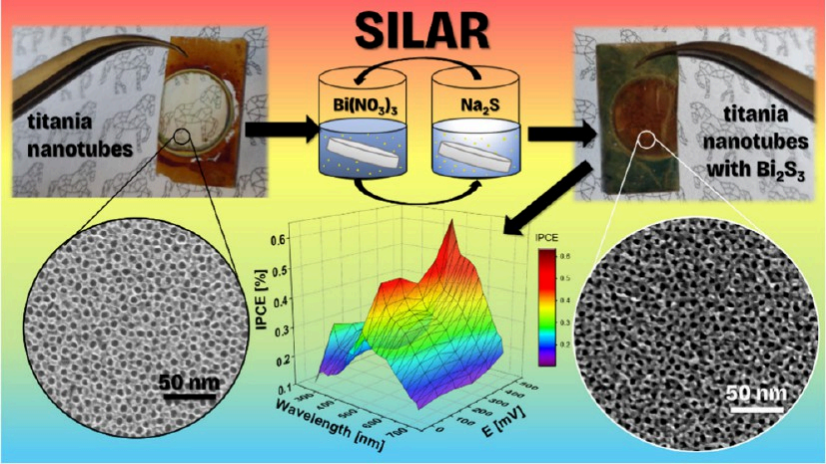

Research on titanium nanotubes modified with metal sulfides, particularly bismuth sulfide (Bi_2_S_3_), aims to create heterostructures that efficiently absorb sunlight and then separate photogenerated charge carriers, thereby enhancing the energy conversion efficiency. This study shows a key role of solvent used for sulfide and bismuth salt solutions used during successive ionic layer adsorption and reaction (SILAR) onto the morphology, structure, and photoresponse of the heterojunction where one element is represented by semitransparent titania nanotubes (gTiNT) and the second is Bi_2_S_3_. Using 2-methoxyethanol and methanol during SILAR, results in remarkably photoactive 3D heterostructure and recorded photocurrents were 44 times higher compared to bare titania nanotubes. Additionally, methanol- and 2-methoxyethanol-based processing allowed uniform deposition of the sulfide, which was not reached for other solvents. XPS studies not only confirm formation of bismuth sulfides but also indicate that Bi_*x*_Ti_*y*_O_*z*_ compound can arise that can affect both stability and photoactivity of the electrode material.

## 1 Introduction

Bismuth sulfide (Bi_2_S_3_) is one of the most interesting materials used in modern photovoltaics and photoelectrode technologies. Its popularity mainly stems from its low band gap (ca. 1.3–1.5 eV) and, as a consequence, high light absorption.^1^ These properties make it useful in optoelectronics, solar energy conversion, infrared radiation detectors, and photocatalysis.^2^ There are many studies in the literature focusing on the deposition of bismuth sulfide on various substrates, such as glass, metal oxides (e.g., TiO_2_, ZnO), metal sulfides, and conductive polymers.^3^ However, porous substrates are becoming increasingly popular due to their ability to enhance photoactivity and charge transport efficiency^4^ due to the developed surface area. Among these, ZnO-based porous substrates are particularly attractive for photoelectrochemical cells and solar energy applications. For example, Bi_2_S_3_ thin films have been coated on ZnO nanorods arrays with varying concentrations of ion precursors using the successive ionic layer adsorption and reaction technique, as demonstrated by Al-Zahrani et al.^5^ Additionally, Zhang et al.^6^ prepared Bi_2_S_3_-modified ZnO nanowires through a combination of electrochemical deposition and successive ionic layer adsorption and reaction technique, showcasing their effectiveness in enhancing photocatalytic properties. Another example of a material is MoS_2_/WS_2_ which serves as an effective catalyst for enhancing photoelectrocatalytic activity. According to Jiang et al.^7^ Bi_2_S_3_ nanorods deposited on MoS_2_/WS_2_ nanosheets using a hydrothermal–electrodeposition strategy promote visible-light utilization and can be considered as a potential candidate for photocatalytic production of H_2_. In our work, we proposed hydrogenated titanium dioxide (TiO_2_) nanotubes as porous and highly ordered substrates for modification. Titanium dioxide nanotubes feature a high specific surface area, good electrical conductivity, and chemical stability, making them ideal candidates for substrates in photoelectrode systems.^8^ Titanium dioxide nanotubes possess a high specific surface area, excellent electrical conductivity, and notable chemical stability, which collectively render them promising substrates for applications in photoelectrode systems.^9^ TiO_2_ nanotubes offer a unique combination of high surface area, porosity, and chemical reactivity, which are beneficial for depositing new materials both inside the hollow interior and on the surface. However, as anodized titania is amorphous; the thermally induced phase transition is required to take advantage of nanotubes as an electrode material. Typically this process is realized in air atmosphere, but annealing in hydrogen provides material exhibiting improved electrical conductivity and reactivity of the material.^10^ This process involves the introduction of hydrogen atoms into the material structure, which in turn leads to the introduction of structural defects, mainly in the form of oxygen vacancies. Those vacancies act as n-type doping, increasing the number of majority charge carriers and improving the electrical properties of the material.^11^ Additionally, the method of annealing the material in a hydrogen atmosphere can force the material to crystallize with less oxygen, creating deeper vacancy states and making it more difficult for the material to relax under atmospheric conditions.^12^ Regardless, under annealing conditions titania exhibit poor photoactivity in visible light, and different strategies are proposed, like nonmetal or metal doping, deposition of metal oxides, or conducting polymer to make the material photoactive.

The modification of titania sometimes requires sophisticated methods and precious equipment, like atomic layer deposition or magnetron sputtering,^13^ making the whole procedure expensive and time-consuming. Realizing this, the SILAR (Successive Ionic Layer Adsorption and Reaction) method has been selected for depositing Bi_2_S_3_ on titanium dioxide nanotubes. This method is notable for its simplicity and low cost, requiring any complex apparatus, but needs step by step optimization.^14^ The SILAR process is based on alternate immersion of the substrate in solutions providing cations and anions, enabling the gradual formation of a thin-film structure through chemical reactions on the surface. This procedure allows for precise control over layer thickness and deposition on substrates with complex morphology by simply changing the concentration of the solution and the number of performed cycles. It is widely used for depositing various semiconductor materials, including sulfides, like zinc sulfide, cadmium sulfide, and bismuth sulfide.^15,16^ Among others, bismuth sulfide is commonly used as a material for constructing photoelectrodes due to its broad light absorption spectrum, favorable photovoltaic properties, and electron transport capabilities. Its use as a photoelectrode is particularly promising in the fields of photocatalysis and solar cells, where its photoactivity can be enhanced by selecting appropriate substrates and deposition methods.^17^ The literature also indicates that the choice of solvent during the deposition of bismuth sulfide can significantly affect its morphology and photoactivity. However, according to the findings of Qiao et al.,^18^ the size of Bi_2_S_3_ nanoparticles is excessively large when synthesized via the solvothermal method. Similar challenges regarding agglomeration and the complete coverage of TiO_2_ nanotubes with Bi_2_S_3_ nanoparticles have been reported by Yu et al.^19^ Furthermore, Caglar et al.^20^ encountered issues with the agglomeration of Bi_2_S_3_, which they addressed by employing a benzoic acid platform. However, this approach also resulted in a reduction of the nanotube dimensions. During the formation of Bi_2_S_3_ on the nanowalls of titania nanotubes, selection of solvent such as deionized water, ethanol, methanol, and 2-methoxyethanol plays a significant role, influencing solvation, volatility, and chemical interactions that ultimately affect material morphology and performance. In this study, deionized water, ethanol, and 2-methoxyethanol were utilized as solvents for the bismuth precursor, while deionized water, ethanol, and methanol were used as solvents for the sulfur precursor. These solvents were chosen to suppress Bi^3+^ hydrolysis and enhanced photoactivity of the formed electrode due to their frequent use in similar syntheses. Their distinct properties allow for controlled reactions and improved deposition uniformity. Among these, 2-methoxyethanol stands out with a high dipole moment (2.36 D) and low vapor pressure (10 hPa),^21^ which promote stable coordination with metals like Bi(NO_3_)_3_, reducing aggregation and ensuring uniform deposition.^22^ Methanol balances solvation and evaporation with its intermediate properties,^21^ while deionized water offers high polarity but risks rapid evaporation.^21^ Ethanol provides stability due to moderate solvation and low vapor pressure,^21^ though it has limited coordination ability compared to 2-methoxyethanol. This systematic analysis highlights the critical influence of solvent properties on the material structure and photoelectrochemical efficiency. Han et al.^23^ identified excessive deposition of Bi_2_S_3_ on TiO_2_ as a critical problem, leading to agglomeration and the formation of recombination centers that diminished photoelectrochemical efficiency. The approach particularly noteworthy to avoid agglomeration may involve the use of the SILAR method, which could facilitate the penetration of bismuth sulfide ions into the interior of the layer composed of highly ordered nanotubes with a hollow interior rather than allowing them to stack on the surface.

To show the importance of solvent selection, in our approach, we focus on the effect of the solvent type used during the SILAR method on the properties of the formed heterostructure, including its structure, morphology, and specifically photoelectrochemical characteristics.^24^ As a platform acting as a sulfide host, highly ordered titania nanotubes formed via anodization of the sputtered Ti layer onto transparent conducting substrate were used. The results gathered during electrochemical and photoelectrochemical studies of titania decorated with bismuth sulfide were crucial in pointing out the importance of precursors’ solvents.

## 2 Materials and Methods

### 2.1 Reagents

ITO glass substrates (S111 thickness, 1.1 mm; ITO thickness, 100 nm; Ossila), acetone (99.5%, Chempur), deionized water (0.08 μS, Hydrolab), ethanol (96%, Chempur), 2-propanol (99.7%, Chempur), methanol (99.8%, Sigma-Aldrich), 2-methoxyethanol (99.0%, Sigma-Aldrich), ethylene glycol (99.5% Chempur), ammonium fluoride (96% Chempur), phosphoric acid (85%, Chempur), bismuth(III) nitrate pentahydrate (98%, Sigma-Aldrich), sodium sulfide nonahydrate (98%, Sigma-Aldrich), and sodium sulfide (99% Chempur).

### 2.2 Electrode Fabrication

The ITO substrates, sized 1.5 × 2 cm^2^, were ultrasonically cleaned sequentially in water, ethanol, acetone, and isopropanol for 15 min each. A thin Ti interlayer was deposited using magnetron sputtering for 30 s at a current of 0.412 A, an argon flow rate of 2 sccm, and a pressure of 2.0 × 10^–3^ Pa (nanoPVD, Moorfield). This initial Ti layer was then thermally treated on a hot plate (PCE-E6000 Series) at 400 °C for 5 min in order to prepare the titania interlayer. Subsequently, a second round of magnetron sputtering was performed for 30 min under the same conditions (0.412 A, 2 sccm argon, and 2.0 × 10^–3^ Pa), resulting in a film thickness of approximately 400 nm. Titania nanotubes were fabricated via an anodization process in a two-electrode system, with the Ti on ITO serving as the anode and a Pt mesh as the cathode. The Ti electrodes were mounted in a custom holder, which exposed only a defined circular area with a diameter of 10 mm to the electrolyte. The electrolyte consisted of 0.27 M NH_4_F, 1 M H_3_PO_4_, 1 vol % H_2_O, and 99 vol % C_2_H_6_O_2_. Anodization was conducted at 23 °C, controlled by a thermostat, and an applied voltage of 40 V for 30 min. The electrodes were then thermally treated at 450 °C for 5 min in rapid thermal annealing (RTA) using a MILA-5000 rapid thermal annealer (Advanced Riko) under a hydrogen atmosphere. The samples were annealed in pure hydrogen (Air Liquide, 4 N) at 450 °C with a heating rate of 5 °C s^–1^. The target temperature was maintained for 5 min, followed by cooling to approximately 50 °C.

Afterward, the SILAR procedure was performed on sample sets using 70 mM bismuth and sulfur ionic solutions with varying numbers of deposition cycles. Prior to the first SILAR cycle, the electrodes were exposed to UV light and ozone for 30 min. Subsequently, modifications involving 10, 20, and 30 SILAR cycles were carried out. One cycle includes immersion of the sample in 70 mM Bi(NO_3_)_3_ in 2-methoxyethanol for 30 min, followed by immersion in 70 mM Na_2_S in methanol for 30 min. The samples were rinsed with the respective organic solvents and dried for 30 s at 75 °C after each cycle. This procedure was repeated for the designated number of cycles, with the immersion time of the subsequent solution reduced to 2 min. In total, three series of samples were prepared with the use of different solvents. Apart from 2-methoxyethanol, deionized water and ethanol were utilized. The final stage involved annealing in an RTA under an argon atmosphere (Air Liquide, 5 N) at 250 °C for 1 h, with a heating rate of 5 °C s^–1^. The cooling method was analogous to that in the case of hydrogenation. The electrodes are marked as gTiNT. The first letter “g” indicates that a glass substrate was used for titanium deposition. Then, an additional letter is added to specify the solvent in which the modification precursor was dissolved: M (methanol and 2-methoxyethanol), W (water), or E (ethanol). Following this, a number indicates the number of SILAR modification cycles.

### 2.3 Sample Characterization

The morphology of the electrodes was investigated by using Schottky field emission scanning electron microscopy (SEM, FEI Company Quanta FEG250) with an ET secondary electron detector at an acceleration voltage of 10 kV. SEM images were analyzed using the ImageJ shareware software. Additionally, morphology analysis was performed using XRTEM. Cross-sections were prepared by a single-beam focused ion beam (FIB) instrument JEOL, JIB-4000, working with a gallium source, and the ex situ data were manually transferred to commercially available Cu grids. Investigations were carried out in a high-resolution transmission electron microscope (HR-TEM) JEOL ARM 200F, working at 200 kV, equipped with an energy-dispersive X-ray spectroscopy (EDX) detector. Structural analysis was conducted via confocal micro-Raman spectroscopy (InVia, Renishaw) with an argon ion laser emitting at 514 nm, operating at 10% of its total power, over the range of 100–1500 cm^–1^. The Raman spectra were studied for all of the sample electrodes before electrochemical measurements. Raman spectroscopy studies were repeated after 128 days to check their stability. The optical properties of the electrodes were checked by using a UV–vis spectrophotometer (Lambda 35 PerkinElmer). The spectra were recorded in the wavelength range of 300–1100 nm. Scan speed was set to 120 nm min^–1^ and slide width to 2 mm. Prior to the measurements, the device was calibrated by using clean ITO glass. The chemical structure was studied by X-ray photoelectron spectroscopy (XPS) using a Thermo Scientific K-Alpha spectrometer. Samples were irradiated with Al Kα = 1486.7 eV X-ray radiation under a pressure of 10^–9^ to 10^–8^ mbar. Survey spectra were recorded by using a pass energy of 150 eV and a step size of 1 eV. High-resolution spectra for oxygen O 1s, titanium Ti 2p, carbon C 1s, bismuth Bi 4f, and sulfur S 2p binding energy regions were obtained with a pass energy of 20 eV and a step size of 0.1 eV.

### 2.4 Electrochemical and Photoelectrochemical Characterization

Electrochemical and photoelectrochemical measurements were carried out in a three electrode system, where the TiO_2_-based electrodes were used as the working electrode, Pt mesh was used as the counter electrode, and Ag/AgCl/0.1 M KCl was used as the reference electrode. The electrolyte was deaerated with argon, and during the measurements, argon flow was kept above the electrolyte. The photoelectrochemical tests were carried out using an AutoLab PGStat 302N potentiostat–galvanostat. Cyclic voltammetry (CV) and linear voltammetry (LV) scans were recorded in deaerated 0.5 M Na_2_SO_4_ solution in the range from −1 to +1 V vs. Ag/AgCl/0.1 M KCl. The CVs were performed with a scan rate of 50 mV s^–1^, while the LVs were performed with a scan rate of 10 mV s^–1^. The LV curves were recorded under chopped visible light illumination provided by a xenon lamp (LOT-Quantum Design GmbH) with a cutoff filter for wavelengths below 420 nm. The light intensity was calibrated by using a silicon reference cell (Rera) to provide 100 mW cm^–2^. The xenon lamp setup functions as a Class AAA solar simulator, characterized by its spectral content (quantified as spectral match), spatial uniformity, and temporal stability. The stability tests when the electrode was exposed to visible light were carried out using the CA technique. In the case of chronoamperometry, the current was recorded at +0.2 V vs. Ag/AgCl/0.1 M KCl lasting 600 s. The incident photon to converted electron (IPCE) ratio and photocurrent values in the function of wavelength and applied potential were measured using a photoelectric spectrometer (Instytut Fotonowy) equipped with a 150 W xenon lamp monochromator and potentiostat. The illumination source was calibrated using a silicon photodiode to calculate light intensities. The curves were recorded at the potential range from 0 to +500 mV and wavelengths from 250 to 700 nm. Measurement points were taken with 100 mV and 20 nm steps. The samples were examined in a three electrode system with a platinum plate as a counter electrode, a sample as a working electrode, and Ag/AgCl/0.1 M KCl as a reference electrode. The built-in mechanical shutter was set to illuminate a sample for 5 s and wait for 5 s before illuminating with a different wavelength. Additionally, CVs were repeated after 128 days to assess the stability of the material.

## 3 Results and Discussion

### 3.1 Structural Characterization

**Figure 1:**
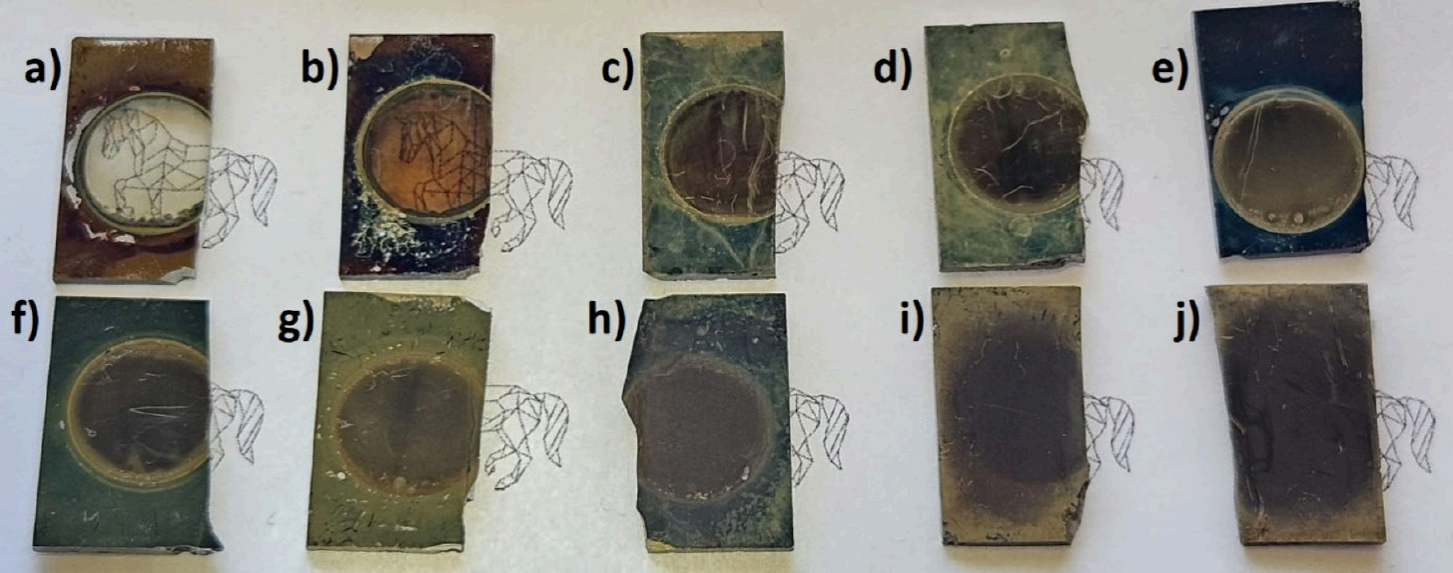
Electrode photography of (a) gTiNT, (b) M10/Bi_2_S_3_/gTiNT, (c) M20/Bi_2_S_3_/gTiNT, (d) M30/Bi_2_S_3_/gTiNT, (e) W10/Bi_2_S_3_/gTiNT, (f) W20/Bi_2_S_3_/gTiNT, (g) W30/Bi_2_S_3_/gTiNT, (h) E10/Bi_2_S_3_/gTiNT, (i) E20/Bi_2_S_3_/gTiNT, and (j) E30/Bi_2_S_3_/gTiNT.

Titania nanotubes exhibit diverse morphological, structural, and electrochemical properties, which are dependent on the parameters of their synthesis and modification. Even a small change in the structure or morphology of these materials has a significant impact on their optical properties, which leads to changes in transparency and color. These differences are clearly visible by the naked eye between the bare titania and those modified using the SILAR method (Figure 1). The first key parameter is the annealing atmosphere. In our previous work^25^ titania nanotubes on a glass substrate, annealed in an air atmosphere, were colorless and completely transparent. Those observations are in agreement with Meyerinka et al.^26^ who also obtained colorless, transparent tubular layers. The effect of hydrogen atmosphere on the properties, including also the appearance of titanium nanotubes, is well-known, but most studies focus on materials synthesized on titanium foil or in the form of powder. Sahoo et al.^27^ reported that titanium nanotubes annealed in hydrogen changed color to darker, and with increasing temperature they became even black. In our case, samples annealed in a hydrogen atmosphere presented a delicate honey glow (Figure 1a). The color changes in the samples on glass are much more subtle and may result from a thin TiO_2_ layer (400 nm) as well as due to the presence of transparent glass/ITO substrate. Another crucial factor influencing the appearance of the samples is the quantity and type of the performed modifications. As the number of cycles increases and the amount of the deposited sulfide grows, the transparency of the samples decreases, regardless of the solvent used. Bi_2_S_3_-modified titanium dioxide nanotubes prepared using methanol and 2-methoxyethanol are semitransparent and have a light amber hue after only 10 SILAR cycles (Figure 1b), while the higher the number of cycles causes the substrate (Figure 1c,d) to darken and lose its transparency. However, when looking directly at these samples, it can be seen that the electrodes in this series retain some transparency. In the cases when water was used to prepare solutions of sulfur and bismuth precursors (Figure 1e,f,g), the effects are as follows: the sample after 10 cycles is slightly transparent, while the samples obtained after 20 and 30 SILAR cycles are nontransparent and have a dark brown color. In contrast, all samples modified with the use of ethanol-based solutions are fully opaque and black. These observations are consistent with the results of Padwal et al.^28^ who also used the SILAR method and observed the change from the transparency through brown and finally black, opaque materials with the increased number of cycles.

**Figure 2:**
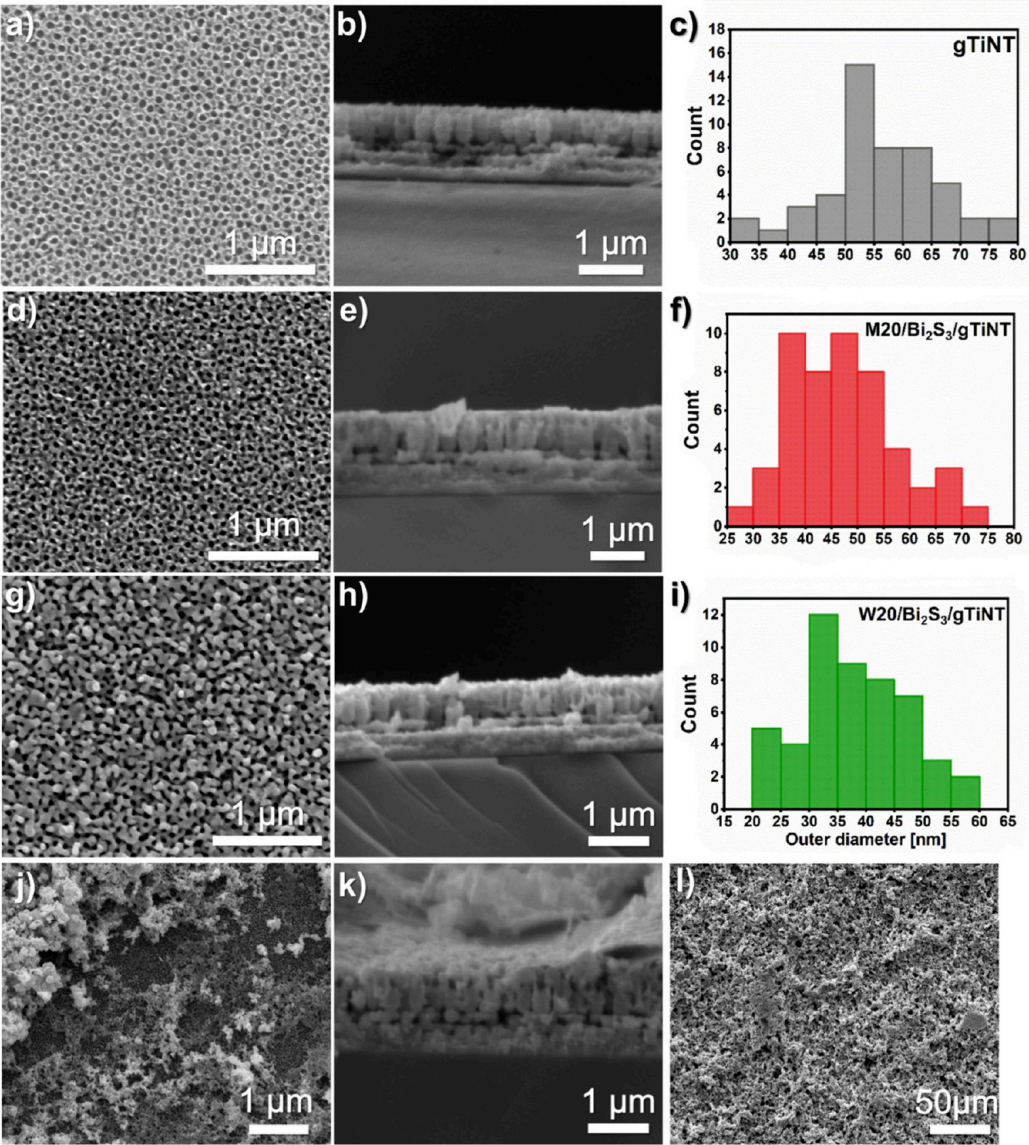
SEM images and size distribution of TiO_2_ nanotubes: SEM images presented in (a, b), (d, e), (g, h), and (j, k, l), while corresponding size distributions shown in (c), (f), and (i) for gTiNT, M20/Bi_2_S_3_/gTiNT, W20/Bi_2_S_3_/gTiNT, and E20/Bi_2_S_3_/gTiNT, respectively.

The SEM images of TiO_2_ nanotubes modified with Bi_2_S_3_, both top view and cross-section, are presented in Figure 2. The selection criterion for the samples for SEM examination was based on the electrochemical and photoelectrochemical results, which are presented and described below. As observed with the naked eye, the alteration of the organic solvent employed in precursor preparation similarly influences the morphology of the samples on the nanoscale. In the process of forming bismuth sulfide using the SILAR method, the empty interiors of the titania nanotubes are filled, and in some samples, it also covers their surface. The appearance of some coverage onto the surface is particularly visible for the sample E20/Bi_2_S_3_/gTiNT. However, the characteristic tubular morphology is preserved for all electrode materials. When 2-methoxyethanol and methanol were used as solvents, a deposited material was observed only inside the nanotubes, while in the case of water-based solution results, sulfide also occupied the top surface of the titania layer. In contrast, for ethanol used as a solvent, one can see unorganized aggregates covering the entire nanotube’s topmost surface. The average height of the nanotubes for all samples reaches 300 ± 10 nm, while the outer diameters for the electrodes gTiNT, M20/Bi_2_S_3_/gTiNT, and W20/Bi_2_S_3_/gTiNT are 55 ± 10, 42 ± 10, and 39 ± 10 nm, respectively, also indicating how the internal diameter decreases after the SILAR treatment. Due to the overloading of sulfide on the surface of the E20/Bi_2_S_3_/gTiNT sample, it was not possible to determine the diameters of the nanotubes.

**Figure 3:**
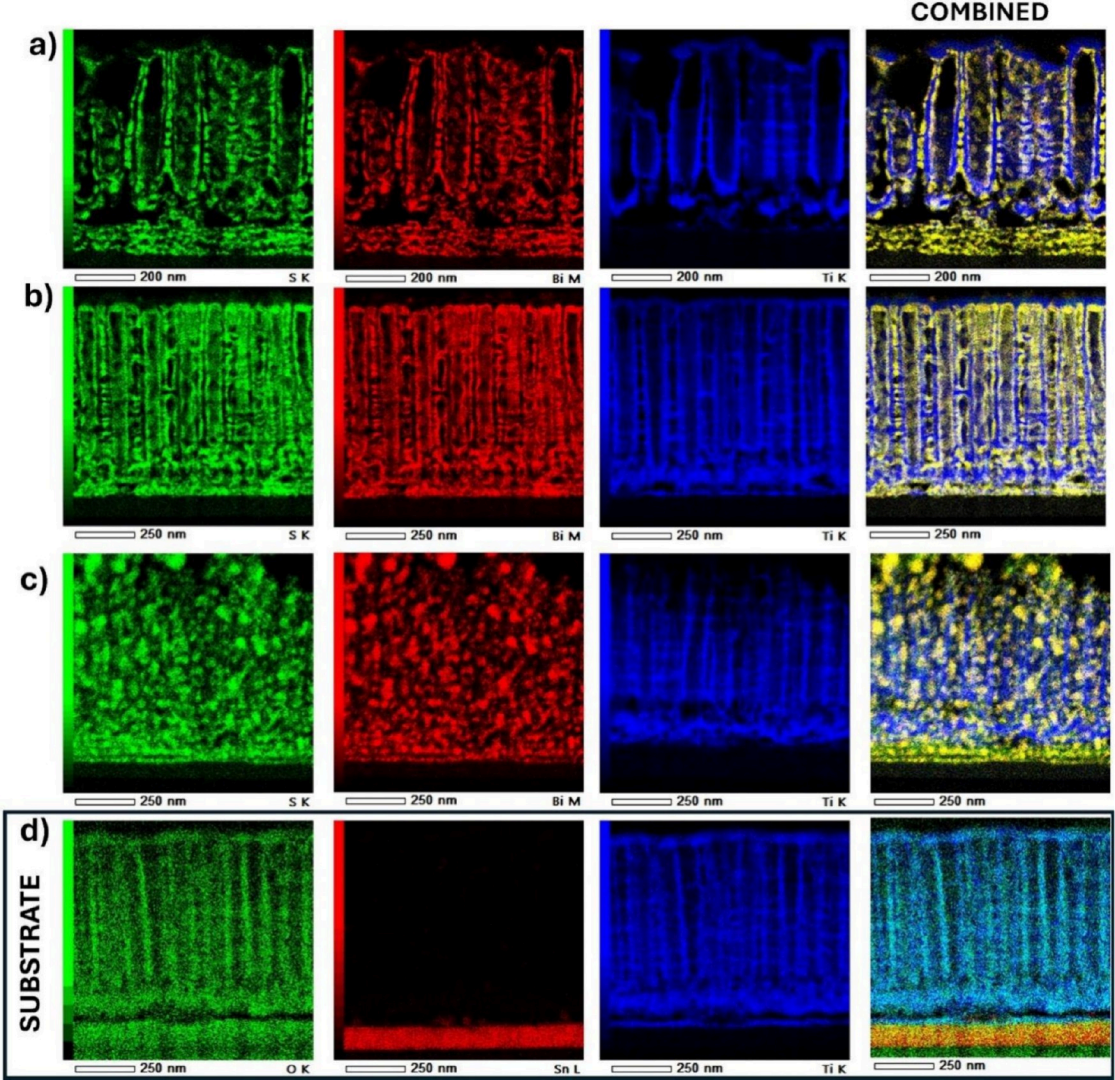
Combined EDX mapping images. The colors in (a) M20/Bi_2_S_3_/gTiNT, (b) W20/Bi_2_S_3_/gTiNT, and (c) E20/Bi_2_S_3_/gTiNT samples represent intensities of the S (green), Bi (red), and Ti (blue) components, respectively. The overlay of the components is shown in the far right panel. (d) Bare substrate for comparison, with O (green), Sn (red), and Ti (blue) color components.

HR-TEM images confirm the morphology of the TiO_2_ samples observed by SEM (Figure 2). Nanotubes are uniformly formed on the ITO substrates without any delamination or detachment. However, more importantly, the EDX mapping analysis, given in Figure 3, shows a homogeneous distribution of Bi and S atoms along the Ti surface and intratubular spaces, except for those observed in Figure 3c. In this case distribution of granular particles in the titania matrix can be observed. As only SEM inspection shows agglomerates on the surface, TEM enables us to see them also at deeper parts of the TiO_2_ platforms. Nevertheless, this shows the high-quality materials in Figure 3a,b and the solvent-dependent nature of the process.

**Figure 4:**
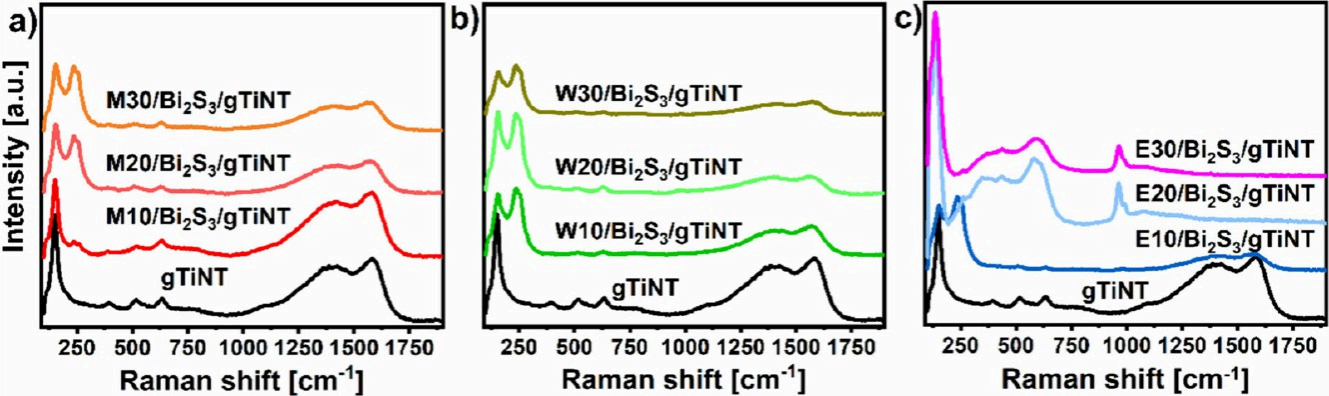
Raman spectra for gTiNT and samples synthesized using (a) 2-methoxyethanol and methanol, (b) water, and (c) ethanol as solvent.

Raman spectra track the types of bonds present for all electrodes in Figure 4. The exact positions of the observed bands are given in Supporting Information Table S1 for all of the series of electrodes. The characteristic peaks of anatase (TiO_2_) that occur at ca. 151, 393, 515, and 635 cm^–1^ are indexed to E_g(1)_, B_1g_, A_1g_, and E_g(3)_, respectively^29^ and are visible for every sample. In both modified and unmodified titania, a shift of the first peak attributed to E_g(1)_ phonon mode compared to the literature data (143 cm^–1^)^29^ can be observed. This slight shift is attributed to the distortion of the crystal lattice by carbon^30^ residues from the anodization process, specifically from ethylene glycol, that could not oxidize and get rid of the sample under the hydrogen atmosphere. The high-intensity signals found at ca. 1375 cm^–1^ (D band) and 1600 cm^–1^ (G band), which also originate from these carbon residues, are responsible for this lattice distortion.^31^ The intensity of those bands decreases with an increasing number of SILAR cycles until they are completely absent for the E20/Bi_2_S_3_/gTiNT and E30/Bi_2_S_3_/gTiNT samples. This is the consequence of the sulfide agglomeration over the surface of the tubular layer, which likely weakens the signal originating from the remaining carbon. It should be underlined that, for all of the modified titania, a double peak with maxima at approximately 237 and 260 cm^–1^, corresponding to the A_g_ and B_1g_ modes of Bi_2_S_3_, respectively, can be observed.^32^ Additionally, an extra shoulder near the band describing the Ti–O bond at approximately 181 cm^–1^ can be seen, which can also be interpreted as the A_g_ mode of Bi_2_S_3_.^32^ In the cases of the E20/Bi_2_S_3_/gTiNT and E30/Bi_2_S_3_/gTiNT electrodes, only a very small band at approximately 260 cm^–1^ is present. However, only for these materials does an additional band appear at 961 cm^–1^. This signal can be associated with nitrogen-containing residues originating from the bismuth precursor Bi(NO_3_)_3_, which is likely adsorbed on the surface in the presence of ethanol as a solvent.^33^ Sample stability results after 128 days are shown in Figure S1, and those spectra show that the structure has been preserved.

### 3.2 Chemical Structure

**Figure 5:**
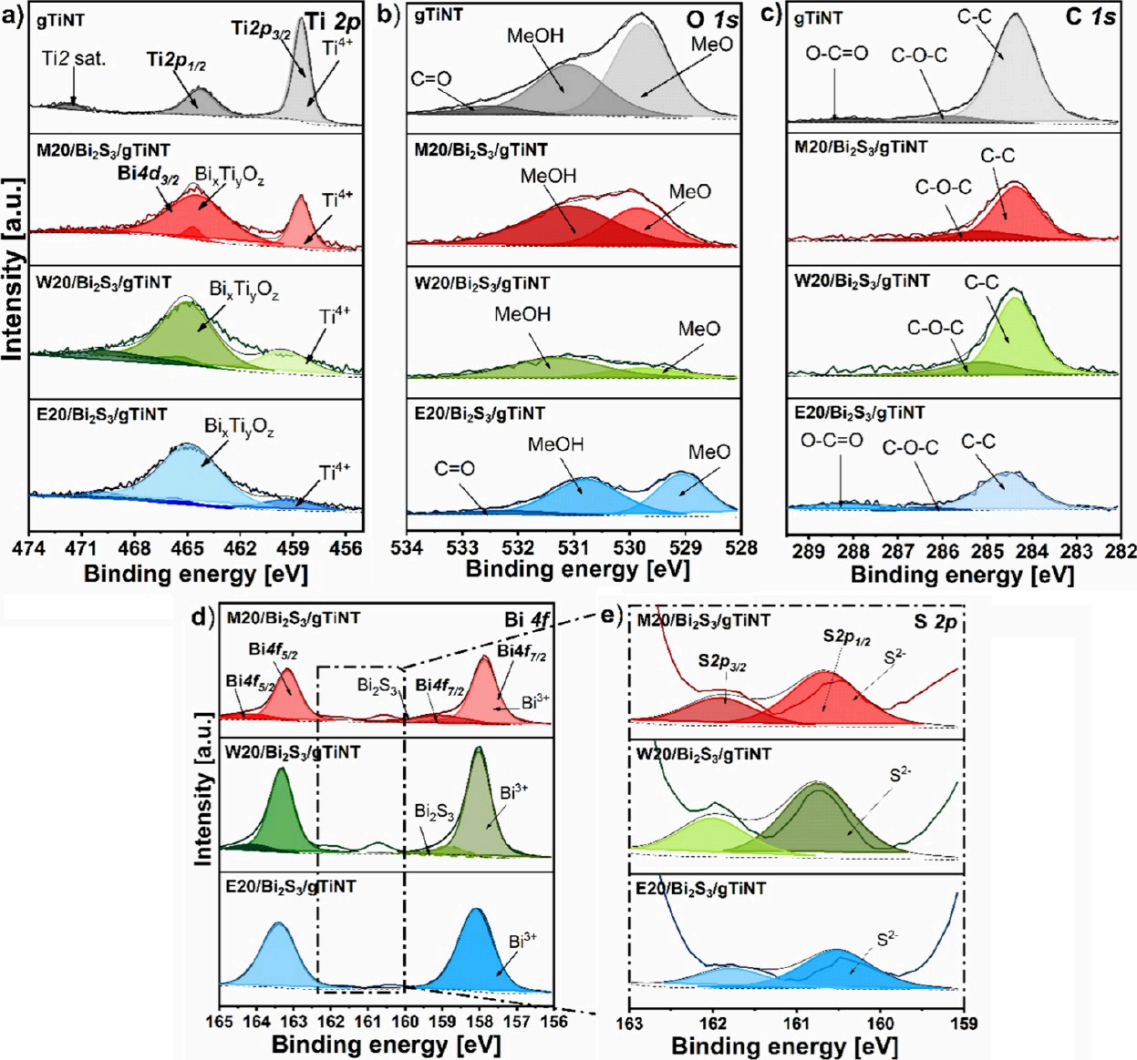
XPS high resolution spectra registered for gTiNT, M20/Bi_2_S_3_/gTiNT, W20/Bi_2_S_3_/gTiNT, and E20/Bi_2_S_3_/gTiNT: (a) Ti 2p, (b) O 1s, (c) C 1s, (d) Bi 4f, and (e) S 2p. Me–Ti or Bi metal.

The chemical states of titanium (Ti 2p, Figure 5a), oxygen (O 1s, Figure 5b), carbon (C 1s, Figure 5c), bismuth (Bi 4f, Figure 5d), and sulfur (S 2p, Figure 5e) were determined by analyzing the results of X-ray photoelectron spectroscopy studies. Detailed interpretation of all recorded spectra was performed based on peak fitting and deconvolution procedures. The obtained binding energies and the percentage of each element and the surface are summarized in Table S2. XPS analysis has been focused on the M20/Bi_2_S_3_/gTiNT electrode due to its high electrochemical activity (described later) and, for comparison purposes, on the electrodes modified by 20 SILAR cycles but using other solvent and gTiNT, without deposited sulfide.

The XPS spectrum for Ti 2p exhibits a doublet of peaks for unmodified sample gTiNT, with binding energies of 458.5 and 464.3 eV. This corresponds to the spin–orbit splitting of Ti 2p_3/2_ and Ti 2p_1/2_. These values were assigned to titanium in the oxidation state of +4, indicating bonding consistent with stoichiometric TiO_2_.^34^ Additionally, the spectrum also contains a distinct charge transfer satellite peak at 471.6 eV. Its origin is a subject of speculation, but the most plausible explanation is strong covalent hybridization between the metal d orbitals and the oxygen p orbitals^35^

Comparing the results obtained for the samples after Bi_2_S_3_ modification, we can see significant changes in the Ti 2p XPS spectrum (Figure 5a). The intensity of the dominant peak decreases and its position slightly shifts toward higher binding energies. These changes are accompanied by a simultaneous increase in the intensity of the second peak at 464.3 eV. Considering the intensity of the changes, the samples can be arranged in the order M20/Bi_2_S_3_/gTiNT < W20/Bi_2_S_3_/gTiNT < E20/Bi_2_S_3_/gTiNT. It should be noted the first sample both peaks are still visibly separated and of comparable intensity. For the next one, the dominance reverses and the overlap of peaks increases, while for the last one, the characteristic peak for Ti^4+^ is barely noticeable. These shifts are consistent with the observations in SEM, where nanotubes are not only filled with Bi_2_S_3_ but also agglomerate on the surface. Since XPS measurement concerns the surface region, an increased dominance of Bi and S elements is identified, which was also observed for the Raman spectra. Taking this into account and the literature data, it was concluded that the spectrum for titanium in heterostructures is due to the presence of signal also for Bi 4d_3/2_ in the same energy range. The characteristic position for Bi 4d_3/2_ is 465.9 eV and thus as a results of Bi_2_S_3_ loading over the nanotube surface, the intensity and width of this peak increase.^36^ Analyzing the XPS spectrum for pure bismuth sulfide performed by Panigrahi et al.,^37^ it can be seen that Bi 4f dominates in the region’s lower binding energies (150–170 eV). For this reason, many works do not present a detailed analysis of the spectrum of Bi 4d. However, during the SILAR processing, a reaction between bismuth and titanium may occur; therefore, the possibility of the formation of a new Bi_*x*_Ti_*y*_O_*z*_ compound should be also taken into account. Referring to the studies describing the XPS results for bismuth titanate (Bi_*x*_Ti_*y*_O_*z*_), it can be seen that the spectra for Ti 2p are analogous to those observed for the samples studied in the presented work.^36^ This may confirm the above assumptions.

The deconvolution of the Bi 4f spectrum was also performed for the titania/sulfide heterostructure (Figure 5d). Two distinct peaks are visible at 157.9 and 163.2 eV, which correspond to the bismuth doublet 4f_7/2_ and 4f_5/2_, respectively. Analogous binding energies of these peaks were also observed by Miniach and Gryglewicz for the Bi_2_S_3_ compound.^38^ For M20/Bi_2_S_3_/gTiNT and W20/Bi_2_S_3_/gTiNT samples, the two dominant peaks are asymmetric with the low-intensity shoulders at 159.2 and 164.6 eV. This pair of signals is close to binding energies also of Bi 4f_7/2_ and 4f_5/2_ in Bi_2_S_3_ and can be assigned to the presence of Bi^3+^ in (BiS_2_)^−^ or [Bi(S_2_O_3_)_3_]^3–^ species.^37^ It was also observed for the Bi_4_Ti_3_O_12_ compound and similarly was attributed to the presence of Bi^3+^.^36^ Depending on the reference, the dominant peak found at 158 eV and others remaining at 159 and 164.2 eV may also indicate the presence of metallic Bi or Bi_2_O_3_, respectively.^39^ In all samples, two additional peaks in the ranges of 163 and 159 eV can be observed, which are correlated with the presence of sulfur given in Figure 5e. The deconvolution for the S 2p spectrum allowed us to determine the position of the peaks as 160.7 and 161.9 eV, representing the spin–orbit pair 2p_3/2_ and 2p_1/2_ in metal sulfides. The localization of the observed signals within S 2p spectra proves the S^2–^ valence state and may be related to the presence of Bi_2_S_3_.^38^

The deconvolution of the O 1s spectrum (Figure 5b) provided three peaks at binding energies of 529.8, 531.1, and 532.5 eV. The first peak is characteristic of the metal–oxygen bonds and can be assigned to the Ti–O bond in the crystalline structure of TiO_2_^40^ and/or Bi–O bond in Bi_*x*_Ti_*y*_O_*z*_ compound as well as in Bi_2_O_3_.^36^ The subsequent maximum corresponds to oxygen vacancies and excess electron density in Ti^41^ or hydroxyl groups present on the surface of the sample.^42^ The last peak, occurring only in the unmodified gTiNT sample and in E20/Bi_2_S_3_/gTiNT, can be attributed to the C=O bond, specifically, to carbonyl groups.

In the case of the C 1s spectrum shown in Figure 5c, three peaks were assigned to binding energy values of 284.5, 286.1, and 288.6 eV. The C 1s peak around 284.5 eV is typically attributed to residual carbon originating from the organic precursor present both in the anodization bath and solutions used during SILAR, which can be adsorbed onto the surface of titania specifically to C–C bonds. The subsequent peaks at 286.1 and 288.6 eV are assigned to C–O–C and O–C=O bonds, respectively, related to ethylene glycol species present in the electrolyte used during the anodization process.^43^

### 3.3 Optical Characterization

**Figure 6:**
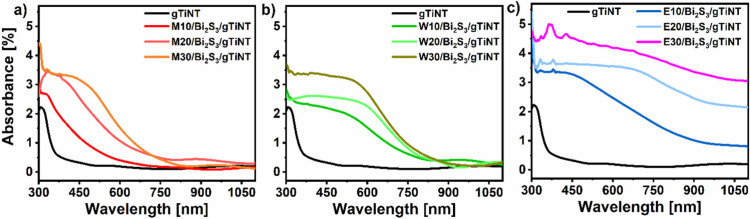
UV–vis reflectance spectra for gTiNT and samples synthesized using (a) 2-methoxyethanol and methanol, (b) water, and (c) ethanol as solvent.

In order to assess the optical properties of the series of fabricated materials, absorption spectra were recorded (Figure 6). The absorption edge for pure titanium nanotubes, labeled as gTiNT, is in the UV region, below 400 nm. For the heterostructures when nanotubes underwent modification with SILAR using 2-methoxyethanol and methanol as solvents for sulfur and bismuth precursors (Figure 6a), the absorption edge is red-shifted compared to the pure nanotube sample. This red shift becomes more pronounced with the increasing number of SILAR cycles. A similar phenomenon is observed for the bismuth sulfide using water as a solvent for sulfur- and bismuth-containing precursors (Figure 6b), where the absorption edge shifts even to ca. 700 nm and undergoes further red-shifting with higher loading of sulfide. Absorbance spectra recorded for heterojunctions when ethanol-based solutions were used (Figure 6c) show light absorption over a broad visible range, being enhanced along with the increased number of SILAR cycles. The characteristic sharp absorption edge of TiO_2_ nanotubes disappears, and the spectra look like the one of pure Bi_2_S_3_.^44,45^ The irregular changes in intensity up to 450 nm for samples where Bi_2_S_3_ was deposited from ethanol solution are probably due to surface inhomogeneity, as confirmed by SEM images (see Figure 2j,k,l and Figure S3e, f)

Taking into account recorded spectra, calculated band gaps (*E*_g_) were determined, and the obtained values are summarized in Table 1 The relationship between the absorption coefficient α and the band gap *E*_g_ of a semiconductor is described by the equation:
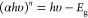
where *h* is Planck’s constant, υ is the light frequency, and *n* takes a value of 0.5 for indirect band gaps and 2 for direct band gaps. In this study, a value of *n* = 0.5 was used in the Tauc method to determine the band gap because the amount of Bi_2_S_3_, the main embedded component, is lower compared to that of TiO_2_ nanotubes. The absorption coefficient α was calculated according to Lambert’s law:
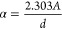
where *A* is the absorbance measured by the UV–vis spectrophotometer and *d* is the thickness of the nanotube and TiO_2_ layer. The Tauc plots for semiconductors with an indirect band gap show (*αhυ*)^2^ vs *hυ*. The band gaps of the samples were determined from the intersection of the extrapolated linear parts of the plots with the energy axis. The value of 3.07 eV is lower than 3.2 eV very often reported in the literature.^46^ However, note that the band gap of nanomaterials can vary depending on their geometric structure. For the modified samples, all band gaps are narrower than those of the reference sample gTiNT (see Table 1), which is expected since the nanotubes were coated with Bi_2_S_3_ exhibiting lower band gap (1.3–1.5 eV).^47^ For the samples modified via SILAR procedure with ethanol as a solvent, the band gap could not be calculated because they absorb light almost in the entire range of visible light. Additionally, the material accumulates at the top surface of the nanotubes, as shown in the SEM images embedded in Figure S1. Considering only the optical properties, better photoelectrochemical performance is anticipated for electrodes coated with Bi_2_S_3_ compared with pure TiO_2_ nanotubes. However, it should be noted that light absorption is one of several criteria playing an important role in efficient photoconversion.

**Table 1:** Energy Gap Value Estimation

ID	energy band gap (eV)
gTiNT	3.07
M10/Bi_2_S_3_/gTiNT	1.41
M20/Bi_2_S_3_/gTiNT	1.81
M30/Bi_2_S_3_/gTiNT	1.29
W10/Bi_2_S_3_/gTiNT	1.29
W20/Bi_2_S_3_/gTiNT	1.15
W30/Bi_2_S_3_/gTiNT	1.19
E10/Bi_2_S_3_/gTiNT	cannot be determined^a^
E20/Bi_2_S_3_/gTiNT	cannot be determined^a^
E30/Bi_2_S_3_/gTiNT	cannot be determined^a^

a Due to the wide absorption spectra.

### 3.4 Electrochemical and Photoelectrochemical Activity

**Figure 7:**
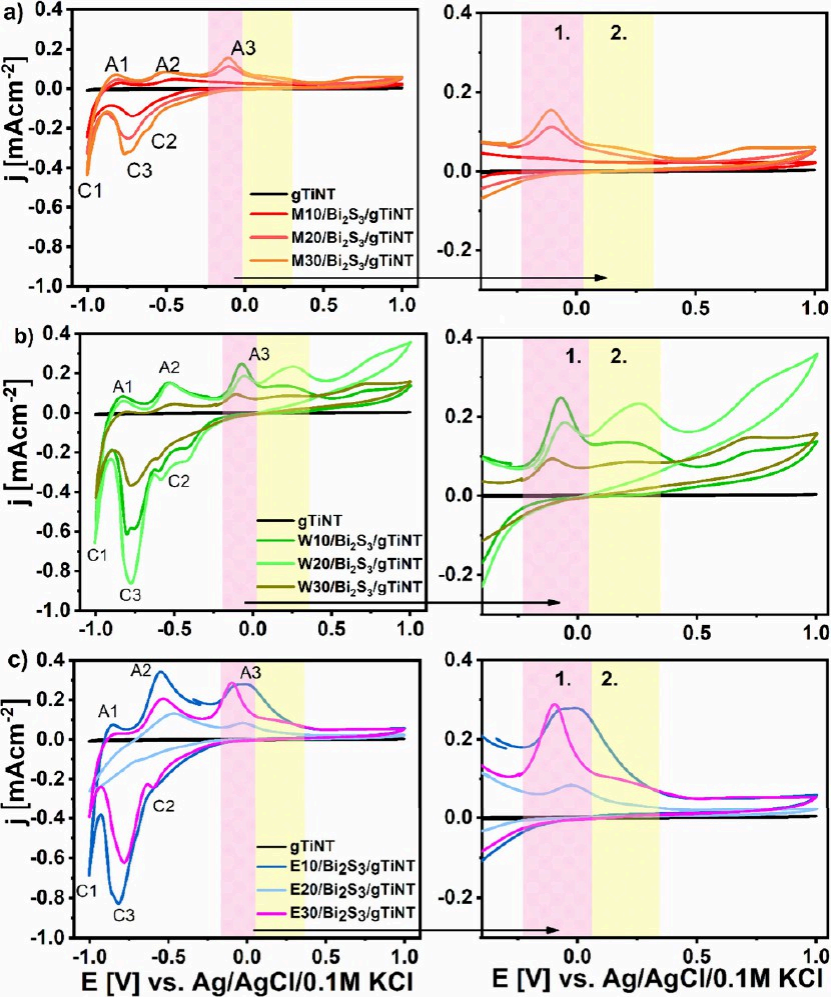
Cyclic voltammetry (CV) curves for gTiNT and samples synthesized using (a) 2-methoxyethanol and methanol, (b) water, and (c) ethanol as a solvent for sulfur and bismuth precursor, registered in 0.5 M Na_2_SO_4_ with scan rate of 50 mV s^–1^.

The investigation of electrochemical activity was initiated by performing cyclic voltammetry in a 0.5 M Na_2_SO_4_ solution. The results are presented in Figure 7 showing prominent changes occurring due to the decoration by sulfide species. Anodic peaks associated with oxidation are marked with the letter A, while cathodic peaks related to reduction are indicated with the letter C with an additional number. The semitransparent sample with hydrogenated titanium nanotubes does not exhibit any specific electrochemical response other than low capacitive currents. A similar behavior of TiO_2_ nanotubes during CV analysis was observed by Li et al.,^48^ who reported that hydrogenated material also showed no faradaic reactions in a neutral electrolyte. In contrast, for all TiO_2_ nanotube electrodes coated with Bi_2_S_3_, a variety of redox reactions were observed. The current peak ca. −0.9 V during anodic scanning (A1) can be attributed to hydrogen desorption, while the peak ca. −1 V during cathodic scanning (C1) is associated with hydrogen adsorption.^49^ It is well-known that, in the cathodic regime, the hydrogen evolution reaction (HER) can occur.^50^ This reaction can be mostly observed when cathodic polarizations performed in acidic or alkaline electrolytes. However, in neutral electrolytes low metal hydride coverage and poor H^+^ donor concentration caused slow HER. Therefore, in our case, those oxidation and reduction peaks marked as A1 and C1 can be correlated with hydrogen coverage on the electrode surface.^51^

Next, the anodic peak (A2) located at ca. −0.5 V and cathodic peak (C2) at ca. −0.6 V registered on the CV curves for all gTiNT electrodes with deposited Bi_2_S_3_ are primarily induced by the adsorption of OH^–^ groups onto the bismuth sulfide species. These groups are formed on the surface after immersion in the neutral electrolyte and electrode polarization:^52^ Bi_2_S_3_ + OH^–^ ⇆ Bi_2_S_3_OH + H_2_O + e^–^. Additionally, the broad signal observed during oxidation may indicate the presence of more than one type of reaction. The double reduction peak can confirm the occurrence of two reactions at similar potentials. Therefore, this signal may occur due to redox reactions associated with changes in the oxidation state from SO_4_^2–^ to SO_3_^4–^.^53^ Subsequently, in order tohave a pronounced showcase of reactions occurring during oxidation (A3) and reduction (C3), they are presented in Figure 7 on the right panel, and the relevant reactions are marked accordingly. As was already discussed in the section dedicated to XPS results, bismuth sulfide or bismuth oxide were detected for all Bi_2_S_3_/gTiNT electrodes. Taking into account electrode composition, we proposed the following mechanism occurring under polarization when the oxidation stage of bismuth can be changed. The recorded oxidation reactions are associated with the increase in the oxidation stage from metallic bismuth to Bi^3+^. This route may involve the disproportionation of Bi^+^ to Bi^2+^ and Bi^3+^, as given by the following reactions:^54^
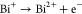
1
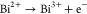
2For samples synthesized using methanol and 2-methoxyethanol as the solvents employed for Bi_2_S_3_ deposition, the oxidation peak marked as A3 is located at −0.1 V. This signal maintains the same shape, but the peak current increases with the multiplication of SILAR cycles performed during modification. However, when water is used as a solvent for precursors, the signal at ca. −0.1 V is the most pronounced for the W30/Bi_2_S_3_/gTiNT sample. For the other two samples in this series, oxidation processes occur in a significantly wider potential range, suggesting the presence of multiple oxidation reactions. Additionally, for the whole set, a notable signal with the maxima located at +0.2 V indicates a change in the oxidation state from Bi^2+^of Bi^3+^ (reaction 2). In the case of samples modified via SILAR when ethanol acted as a solvent, similar behavior is observed. The E10/Bi_2_S_3_/gTiNT electrode exhibits a very broad electrochemical activity from ca. −0.1 to +0.3 V, which likely includes contributions from all three reactions. The E20/Bi_2_S_3_/gTiNT sample shows the strongest signal being the outcome of the oxidation of Bi^1+^ to Bi^2+^ (reaction 1). The electrode modified with the highest number of cycles, that is 30, shows the strongest signal, namely, the highest peak currents recorded oxidation of Bi^2+^ to Bi^3+^.^54^ These variations may arise from the distinct morphologies of the nanotubes resulting from the SILAR process and contact with different solvents during sulfide formation onto titania nanowalls. Such broad signals indicate the occurrence of redox reactions involving the oxidation of metallic bismuth and the reduction of bismuth oxides.^55^ Furthermore, the intercalation of Na^+^ ions can contribute to the oxidation A3 peak and reduction C3, respectively. Intercalation can be described by the following equation:^56^ Bi_2_S_3_ + *x*Na^+^ + *x*e^–^ → Na_*x*_Bi_2_S_3_. Such a phenomenon is observed around 0 V, indicating that the sodium ions are incorporated into the structure of the modified sulfide material. The extensive signal observed within the potential range of −0.2 to +0.3 V indicates a complex reaction mechanism that likely encompasses multiple processes. These processes may include the insertion of sodium ions (Na^+^) into both the sulfide matrix and the tubular layer, followed by their subsequent release. Additionally, interactions between the electrode surface and the surrounding electrolyte may further contribute to the observed electrochemical behavior.^56^ Sample stability results after 128 days are shown in Figure S2.

**Figure 8:**
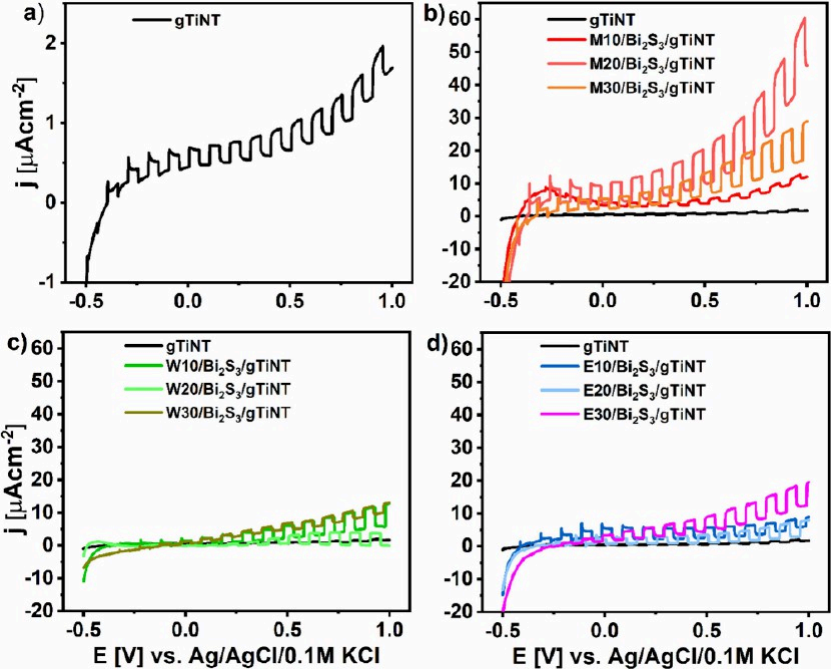
Linear voltammetry curves for (a) gTiNT and samples synthesized using (b) 2-methoxyethanol and methanol, (c) water, and (d) ethanol as solvent for bismuth and sulfur precursors under visible light illumination registered in 0.5 M Na_2_SO_4_ with the scan rate of 10 mV s^–1^.

To obtain information about charge transfer on electrodes, LV curves under chopped visible light illumination (Figure 8) were recorded for all of the electrodes. In the cathodic potential range from −0.5 to −0.4 V, no photocurrents were detected, with the current density (*j*) remaining negative and stable. Photocurrent generation is initiated when the potential exceeds −0.4 V and persists steadily up to 1 V. Such behavior suggests that, under illumination, photogenerated holes reaching the electrode/electrolyte interface become trapped, leading to their recombination of photogenerated carriers. Upon completion of the material’s exposure to irradiation, the trapped holes undergo oxidation, leading to the emergence of a distinct cathodic peak.^29,30^ Under the anodic polarization, the recorded photocurrents increase or reach the threshold value depending on the material; however, cathodic spikes are no longer detected. Along with the applied potential, the strong electric field formed in the double layer acts as a driving force toward the neutralization of holes and supports the separation of photogenerated charge carriers.^59^ The run of the LV curves for all electrodes is similar; however, huge differences in achieved photoactivity can be found. The highest photocurrent value of 15.5 μA cm^–2^ at +0.6 V vs. Ag/AgCl/0.1 M KCl was recorded for M20/Bi_2_S_3_/gTiNT. This is 44 times higher than the photocurrent measured at the same potential for pure nanotubes (0.35 μA cm^–2^). The photocurrent enhancement that has been reached for the M20/Bi_2_S_3_/gTiNT heterostructure significantly surpasses results reported by other researchers. For instance, Hreo et al.^60^ reported a 4-fold increase in photocurrent between Bi_2_S_3_-modified nanotubes and pristine titania, while Freitas et al.^61^ achieved a 3-fold increase under similar conditions. Additionally, Wu et al.^62^ observed a 14-fold increase in photocurrent between their most optimized modified sample and pure TiO_2_ nanotubes, which is still lower than the differences observed in our study. Furthermore, Wu et al.^63^ achieved photocurrent enhancements of nine and eight owing to the Bi_2_S_3_ deposition, again falling short of the improvements demonstrated here. As is obvious, the shift of the absorption edge toward longer wavelengths, as observed in the absorption spectra of Bi_2_S_3_-decorated nanotubes, can be treated as a major factor enhancing the generated photocurrent. However, in this case, recombination processes may occur. Excitons cannot be separated, causing the material to behave more like a conductor than a semiconductor. There is also the possibility of exciton dissociation or exciton quenching. Thus, a key role is played here by solvents used during the SILAR process, namely, 2-methoxyethanol and methanol, leading to the uniform and effective coating of titanium nanotubes with Bi_2_S_3_. In consequence 3D heterostructure exhibits significantly better photoelectrochemical performance compared to samples fabricated according to the ethanol- or water-based routes.

The higher solubility of the precursor in 2-methoxyethanol and methanol allows for a uniform distribution of ions across the nanoporous titania substrate. As a result, homogeneous sulfide layers are achieved, which enhances both optical properties and photoactivity.^64^ For instance, bismuth nitrate pentahydrate dissolves well in 2-methoxyethanol, which is frequently used in the synthesis of nanostructured bismuth compounds. Although specific solubility values for 2-methoxyethanol are not always reported, its ability to dissolve compounds like bismuth nitrate makes it a valuable solvent in nanomaterial fabrication.^65^ Similarly, sodium sulfide nonahydrate demonstrates solubility values of 5.1 g/(100 g) in methanol, further supporting its role in producing uniform sulfide layers.^66^ Methanol and 2-methoxyethanol, functioning as solvents for bismuth and sulfide salts, may mitigate the oxidation processes on the surface of titanium nanotubes. This reduction in oxidation contributes to the preservation of a more stable Bi_2_S_3_ phase on the surface, resulting in an enhanced photocurrent.^15^ Water is relatively more polar, which can lead to hydrolysis of precursors and faster oxidation, which in some cases is not favorable for maintaining the stability of sulfide layers on the nanoporous surface of titania.^67^ In fact, the presence of water can promote the uncontrolled nucleation of molecules and the formation of inhomogeneous layers. On the other hand, ethanol, although less polar than water, is often used to stabilize metal ions, such as bismuth ions, but its weaker ability to dissolve precursors can limit the control over the deposition of uniform layers. In SILAR procedures utilizing ethanol and water, there is a pronounced tendency for the formation of inhomogeneous layers, which may hinder the attainment of stable Bi_2_S_3_ phases on the titania surface.

**Figure 9:**
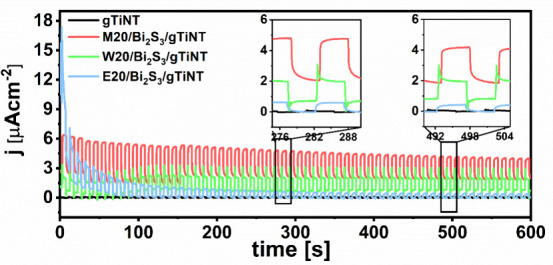
Chronoamperometry curves for gTiNT, M20/Bi_2_S_3_/gTiNT, W20/Bi_2_S_3_/gTiNT, and E20/Bi_2_S_3_/gTiNT recorded at +0.2 V vs. Ag/AgCl/0.1 M KCl under periodical dark/vis conditions.

The stability of the photoelectrochemical performance of M20/Bi_2_S_3_/gTiNT, as the most photoactive electrode, was verified using chronoamperometry measurements conducted at +0.2 V vs. Ag/AgCl/0.1 M KCl (Figure 9). For comparison, results for samples fabricated using other solvents for bismuth and sulfur precursors but preserving the same number of SILAR cycles, as well as bare titania, were also included. CA results for the remaining samples can be found in the Supporting Information. As expected, the gTiNT sample shows a very low but stable current throughout the entire stability test. In the case of the W20/Bi_2_S_3_/gTiNT electrode, long-term stability is also noted with slight changes in current density characterized by high anodic and cathodic spikes at each change in illumination indicating high charge recombination.^57,58^ For the E20/Bi_2_S_3_/gTiNT electrode one can observe a decrease in photocurrent density over time, although it maintains an almost square shape. The last one in the tested set, namely, M20/Bi_2_S_3_/gTiNT, initially loses some photoactivity but then stabilizes and keeps a steady but the highest photocurrent value among all modified titania. The shape of the chronoamperometry curve recorded during the periodic exposition of the electrode to visible light suggests lowered charge recombination compared to the electrodes where Bi_2_S_3_ was deposited from aqueous solution. These results clearly indicate that to achieve improved photoelectrochemical performance, when holes trapped at the electrode/electrolyte interface can be neutralized even at the potential of +0.2 V, methanol and 2-methoxyethanol should be used as organic solvents for sulfur and bismuth precursors.^59^ In comparison to nanotubes modified via a procedure including ethanol as a solvent, these materials show higher photocurrent and better long-term stability.

**Figure 10:**
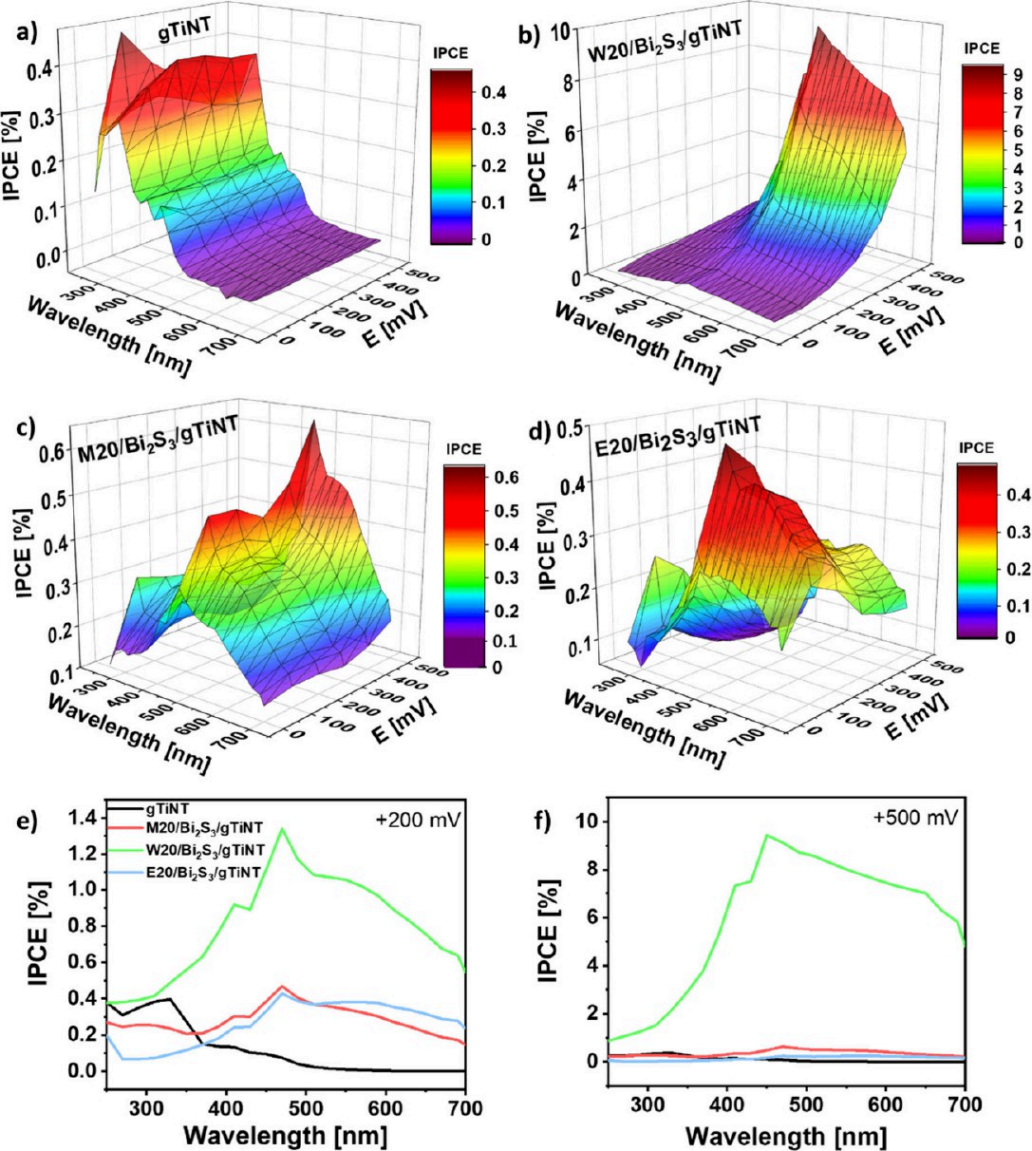
IPCE results for (a) gTiNT, (b) W20/Bi_2_S_3_/gTiNT, (c) M20/Bi_2_S_3_/gTiNT, and (d) E20/Bi_2_S_3_/gTiNT and the respective 2D figures (e) + 200 mV and (f) + 500 mV.

The incident photocurrent conversion efficiency (IPCE) measurement is essential for detailed studies regarding the limiting factors of photoelectrode performance. Figure 10 shows IPCE registered for the gTiNT, W20/Bi_2_S_3_/gTiNT, M20/Bi_2_S_3_/gTiNT, and E20/Bi_2_S_3_/gTiNT electrodes. As shown in Figure 10a, the gTiNT electrode has the highest photon-to-current efficiency in the wavelength range from 250 to 370 nm, which is typical for UV active titania and is in accordance with our previous work.^68^ Furthermore, annealing in a hydrogen atmosphere can result in enhanced activity in visible light which can be seen as a second range on IPCE from 370 to 490 nm. However, since we have transparent nanotubes and the semiconductor layer is thin, this change is less significant compared to the hydrogenated nanotubes produced on Ti foil. In the case of Bi_2_S_3_ modified TiO_2_ nanotubes; the maximum of IPCE shifts toward higher wavelengths. This phenomenon is related to better light capture as the shift of the absorption edge to ca. 700 nm (Figure 6) occurs. According to Arshad et al.,^69^ Bi_2_S_3_ nanostructures are characterized by the maximum absorption wavelength from ca. 450 to 480 nm which is responsible for improved photoactivity of bismuth sulfide, as observed in our samples. The highest IPCE value of 9.4% at +0.5 V vs. Ag/AgCl/0.1 M KCl was registered for the W20/Bi_2_S_3_/gTiNT electrode under illumination with a wavelength of 450 nm (Figure 10b). This result is not consistent with the LV and CA measurements, where the M20/Bi_2_S_3_/gTiNT sample achieved the highest photocurrent value. However, it should be underlined that LV and CA measure photocurrent during the whole range of visible light illumination, whereas IPCE during illumination by a particular wavelength. Moreover, the IPCE measures the theoretical potential of the material to convert photons into charges, while actual photocurrents from voltamperometry measurements can be lowered due to charge recombination processes.^70^ Such an explanation is in accordance with the presence of high anodic and cathodic spikes registered during CA measurement only for the W20/Bi_2_S_3_/gTiNT electrode (Figure 9). Considering the bismuth sulfide modified electrodes examined in this study, specifically W20/Bi_2_S_3_/gTiNT, M20/Bi_2_S_3_/gTiNT, and E20/Bi_2_S_3_/gTiNT, only the W20/Bi_2_S_3_/gTiNT electrode (Figure 10b) exhibits an increase in incident photon-to-current efficiency that commences upon exceeding a potential of +0.2 V. This phenomenon can be correlated with CV results (Figure 7b), where oxidation processes marked as A3 occur in a wide potential range. It can be claimed that when the potential exceeds +0.2 V, the Bi^3+^ compounds responsible for the significant increase in IPCE start to form on the W20/Bi_2_S_3_/gTiNT electrode surface.

## 4 Conclusions

Research on titanium nanotubes modified with metal sulfides, particularly bismuth sulfide, aims to create heterostructures that efficiently absorb sunlight and separate photogenerated charge, thereby enhancing the energy conversion efficiency. This study investigated the importance of precursor solvent on the quality and properties of the formed heterostructure in which bismuth sulfide and titanium dioxide were brought together. Titanium dioxide nanotubes were formed via anodization of Ti layer sputtered on ITO glass substrate and served as a semitransparent hierarchical hosting platform, with bismuth sulfide acting as a decorative compound. The resulting Bi_2_S_3_/gTiNT heterojunctions exhibited increased photoactivity under visible light compared to that of bare titanium nanotubes. It was found that nanotubes coated with bismuth sulfide using 2-methoxyethanol and methanol as solvents demonstrated superior photoelectrochemical activity and long-term stability compared with those produced with ethanol or water. Additionally, methanol and 2-methoxyethanol allowed for uniform modification of titanium nanotubes via the SILAR method, as confirmed by SEM analysis. Raman spectroscopy and XPS revealed the formation of bismuth sulfide within the tubular nanostructure, and it could be stated that also the new Bi_*x*_Ti_*y*_O_*z*_ compound can be formed and played a role in both material stability and its photoactivity.

## Supporting Information Available

Raman spectra and cyclic voltammetry (CV) curves performed after 128 days, Raman band positions, SEM images, and percentage contribution (at. %) and binding energy of core levels (eV) for the set of fabricated materials (PDF)
