# Supplementary material for: Solvent Selection as a Key Factor in the Performance of Semitransparent Heterojunctions Composed of Hydrogenated Nanotubes and Bismuth Sulfides
Source: ACS Appl Mater Interfaces. 2025 Jan 16;17(4):6728–41. doi: 10.1021/acsami.4c18233 (PMC11788988; doi:10.1021/acsami.4c18233)
Supplement: Supplementary file 1 — am4c18233_si_001.pdf [file am4c18233_si_001.pdf]

Supporting Information

Solvent Selection as a Key Factor in the Performance of Semi-Transparent Heterojunctions Composed of Hydrogenated Nanotubes and Bismuth Sulfides

Stefania Wolff[a,b*], Wiktoria Lipińska[b], Justyna Gumieniak[c], Agnieszka Kramek[c], Karol Załęski[d], Emerson Coy[d], Natalia A. Wójcik[a], Katarzyna Siuzdak[b]

[a] *Advanced Materials Centre and Division of Electrochemistry and Surface Physical Chemistry, Institute of Nanotechnology and Materials Engineering, Gdańsk University of Technology, 11/12 G. Narutowicza Street, 80-233 Gdańsk, Poland*

[b] *Centre for Plasma and Laser Engineering, Institute of Fluid-Flow Machinery, Polish Academy of Sciences, 14 Fiszera Street, 80-231 Gdańsk, Poland*

[c] *The Faculty of Mechanics and Technology, Rzeszów University of Technology, Kwiatkowskiego 4 Street, 37-450 Stalowa Wola, Poland*

[d] *NanoBioMedical Centre, Adam Mickiewicz University, Wszechnicy Piastowskiej 3, 61-614 Poznan, Poland*

**Corresponding author:* stefania.wolff@pg.edu.pl

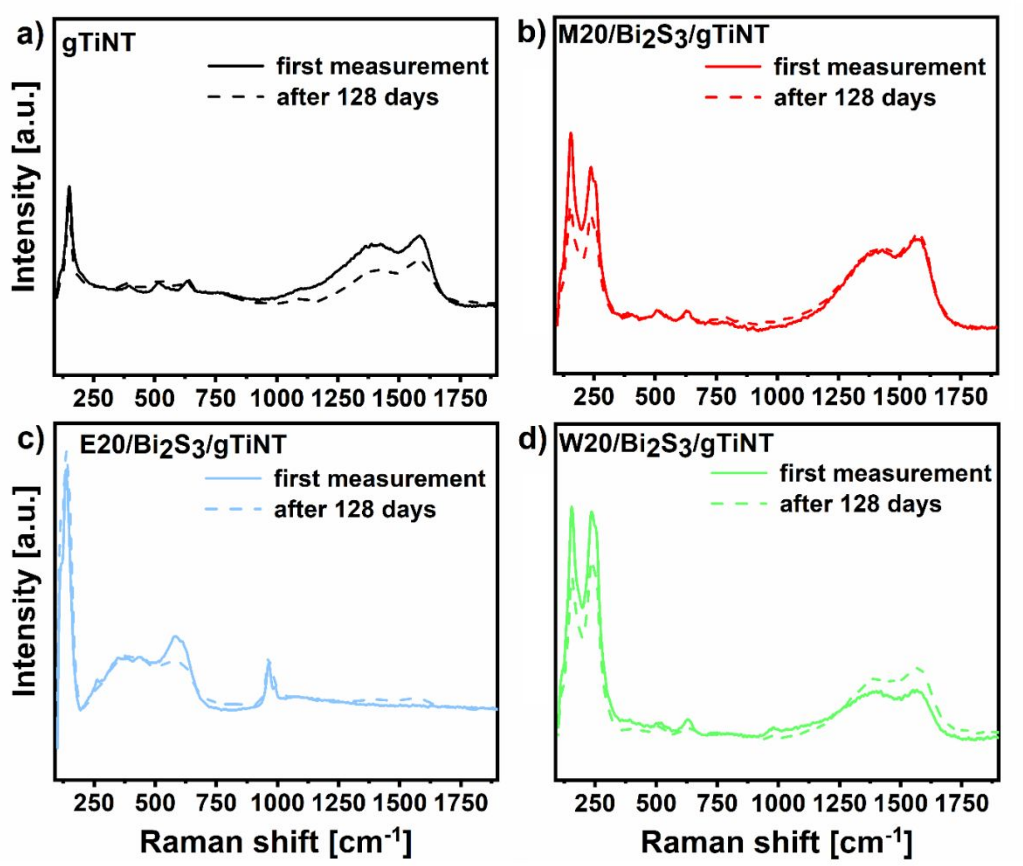

Figure S1 Raman spectra performed after 128 days again for a) gTiNT and samples synthesized using: b) 2-methoxyethanol and methanol, c) ethanol and d) water as solvent for sulfur and bismuth precursor.

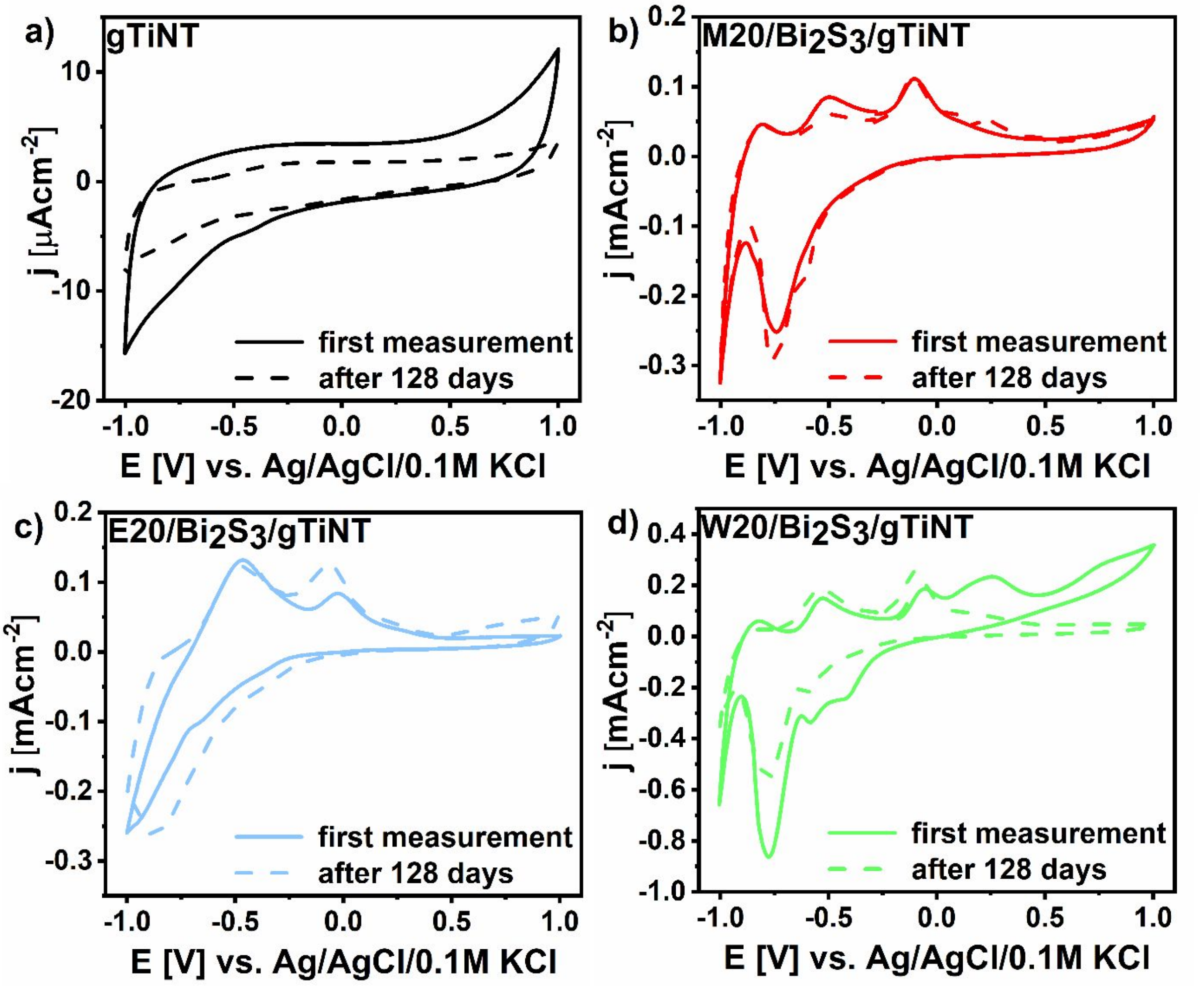

Figure S2 Cyclic voltammetry (CV) curves performed after 128 days again for a) gTiNT and samples synthesized using b) 2-methoxyethanol and methanol, c) ethanol and d) water as a solvent for sulfur and bismuth precursor, registered in 0.5 M Na_2SO_4 with scan rate of 50 mVs^{-1}.

Table S1. Raman bands positions for the set of fabricated materials.
The position is given in cm^{-1}.

IDs	Ti-O (Eg_1)[43]	Bi-S[46]	Bi-S[46]	Ti-O (B_{1g})[43]	Ti-O (A_{1g})[43]	Ti-O (E_{g3})[43]	N-(OH)[47]	C (D band)[45]	C (G band)[45]
gTiNT	151	-	-	393	515	635	-	1375	1590
M10/Bi_2S_3/gTiNT	152	237	260	390	515	633	-	1390	1585
M20/Bi_2S_3/gTiNT	154	237	258	390	515	632	-	1400	1583
M30/Bi_2S_3/gTiNT	154	237	257	390	515	632	-	1400	1582
W10/Bi_2S_3/gTiNT	154	235	257	390	515	630	-	1392	1575
W20/Bi_2S_3/gTiNT	154	235	256	390	515	630	-	1392	1575
W30/Bi_2S_3/gTiNT	156	235	256	390	515	629	-	1400	1575
E10/Bi_2S_3/gTiNT	151	237	256	390	515	629	-	1380	1580
E20/Bi_2S_3/gTiNT	137	-	260	370	455	600	961	-	-
E30/Bi_2S_3/gTiNT	137	-	260	375	455	601	961	-	-

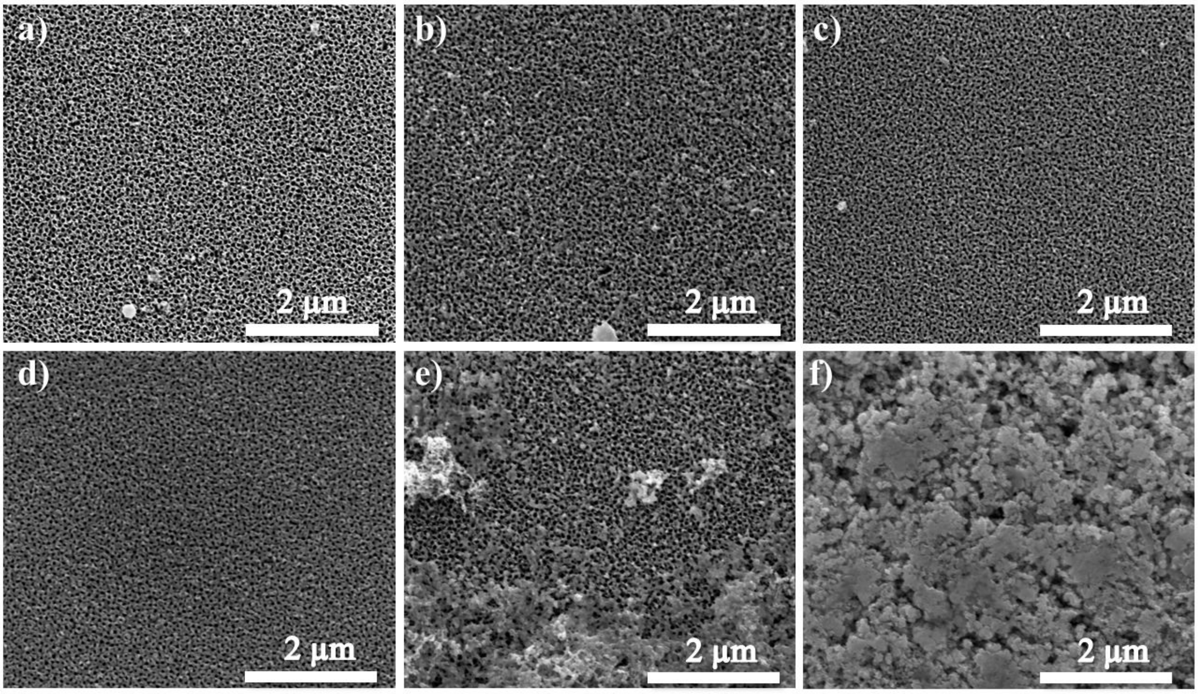

Figure S3 SEM images of a) M10/Bi_2S_3/gTiNT, b) M30/Bi_2S_3/gTiNT,c) W10/Bi_2S_3/gTiNT, d) W30/Bi_2S_3/gTiNT, e) E10/Bi_2S_3/gTiNT and f) E30/Bi_2S_3/gTiNT.

Table S2 Percentage contribution (in at.%) and binding energy of core levels (in eV) of gTiNT and modification samples.

Sample	Peak	Component	BE eV	Atomic %
**gTiNT**	Ti *2p*$_{3/2}$	Ti^{4+}	458.5	10.95
	O *1s*	C=O	532.5	2.04
	O *1s*	MeOH	531.1	15.64
	O *1s*	MeO	529.8	21.08
	C *1s*	O-C=O	288.6	1.29
	C *1s*	C-O-C	286.1	3.32
	C *1s*	C-C	284.5	45.68
M20/Bi_2S_3/gTiNT	Bi *4d*$_{3/2}$	$Bi_xTi_yO_z$	464.4	3.64
	Ti *2p*$_{3/2}$	Ti^{4+}	458.5	5.71
	S *2p3/2*	S^{2-}	160.7	20.34
	Bi 4f$_{7/2}$	Bi_2S_3	159.1	2.37
	Bi 4f$_{7/2}$	Bi^{3+}	157.9	13.98
	O *1s*	MeOH	531.1	10.58
	O *1s*	MeO	529.9	12.06
	C *1s*	C-O-C	285.34	11.15
	C *1s*	C-C	284.5	21.90
W20/Bi_2S_3/gTiNT	Bi *4d*$_{3/2}$	$Bi_xTi_yO_z$	465.0	4.34
	Ti *2p*$_{3/2}$	Ti^{4+}	459.5	2.23
	S *2p*$_{3/2}$	S^{2-}	160.8	26.83
	Bi *4f*$_{7/2}$	Bi_2S_3	158.9	3.77
	Bi *4f*$_{7/2}$	Bi^{3+}	158.0	18.50
	O *1s*	MeOH	531.3	3.93
	O *1s*	MeO	529.8	3.03

	C *1s*	C-O-C	285.4	10.96
	C *1s*	C-C	284.5	27.55
E20/Bi₂S₃/gTiNT	Bi $4d_{3/2}$	$Bi_xTi_yO_z$	464.8	4.64
	Ti $2p_{3/2}$	Ti^{4+}	459.3	1.76
	S $2p_{3/2}$	S^{2-}	160.5	23.35
	Bi $4f_{7/2}$	Bi^{3+}	158.1	20.42
	O *1s*	C=O	532.2	2.92
	O *1s*	MeOH	530.8	9.89
	O *1s*	MeO	529.1	11.71
	C *1s*	O-C=O	288.3	4.87
	C *1s*	C-O-C	286.4	4.36
	C *1s*	C-C	284.6	16.09
